# Psychological Distress and Health-Related Quality of Life in Romanian Adults with Diabetes Mellitus: A Cross-Sectional Study

**DOI:** 10.3390/healthcare14020158

**Published:** 2026-01-08

**Authors:** Lucia Bubulac, Carmen Gabriela Dobjanschi, Mirela Zivari, Constantin Erena, Viorica Tudor, Arsenie Dan Spînu, Gabriela Cornelia Muşat, Claudia Florina Bogdan-Andreescu, Emin Cadar, Cristina-Crenguța Albu

**Affiliations:** 1 Department of Family Medicine, Faculty of Medicine, “Carol Davila” University of Medicine and Pharmacy, 020021 Bucharest, Romania; lucia.bubulac@umfcd.ro; 2 Discipline of Diabetes, Nutrition and Metabolic Diseases, Faculty of Medicine, “Carol Davila” University of Medicine and Pharmacy, 020021 Bucharest, Romania; carmen.dobjanschi@umfcd.ro; 3 Department of Psychology and Educational Sciences, Faculty of Psychology, Ecological University of Bucharest, 061341 Bucharest, Romania; 4 Department of Psychology, Emergency University Hospital, 050098 Bucharest, Romania; 5 Internal Medicine Department, Faculty of Medicine, “Carol Davila” University of Medicine and Pharmacy, 020021 Bucharest, Romania; constantin.erena@umfcd.ro; 6 Bucharest Emergency Clinical Hospital, 014461 Bucharest, Romania; 7 Integrated Outpatient Department, “Prof. Dr. Agrippa Ionescu” Clinical Emergency Hospital, 011356 Bucharest, Romania; tudorviorica_med@yahoo.com; 8 Department 3, Faculty of Medicine, “Carol Davila” University of Medicine and Pharmacy, 020021 Bucharest, Romania; 9 “Dr. Carol Davila” Central Emergency University Military Hospital, 010825 Bucharest, Romania; 10 Department II, Faculty of Dentistry, “Carol Davila” University of Medicine and Pharmacy, 020021 Bucharest, Romania; gabriela.musat@umfcd.ro; 11 Department of Speciality Disciplines, “Titu Maiorescu” University, 031593 Bucharest, Romania; claudia.andreescu@prof.utm.ro; 12 Faculty of Pharmacy, “Ovidius” University, 900470 Constanța, Romania; emin.cadar@365.univ-ovidius.ro; 13 Department of Genetics, Faculty of Dentistry, “Carol Davila” University of Medicine and Pharmacy, 020021 Bucharest, Romania; cristina.albu@umfcd.ro

**Keywords:** diabetes mellitus, anxiety, depression, perceived stress, health-related quality of life, EQ-5D, psychological burden, chronic disease

## Abstract

Background: Psychological distress is increasingly recognized as an important determinant of health-related quality of life (HRQoL) in adults with diabetes mellitus (DM). However, data quantifying this burden in Eastern European populations remains limited. Objectives: This study aimed to assess psychological distress—defined as anxiety, depressive symptoms, or perceived stress—and HRQoL among Romanian adults with DM compared with healthy controls, and to explore potential differences between diabetes subtypes. Methods: A cross-sectional study was conducted, including 400 adults (201 with DM and 199 healthy controls). Psychological distress was evaluated using the State–Trait Anxiety Inventory (STAI-Y1, STAI-Y2), Beck Depression Inventory (BDI), and Holmes–Rahe Stress Scale, while HRQoL was assessed using the EQ-5D Visual Analogue Scale. Group comparisons were performed using appropriate parametric or non-parametric tests, with additional multivariable analyses adjusting for age, sex, and body mass index. Results: Adults with diabetes exhibited significantly higher levels of anxiety, depressive symptoms, and perceived stress, and lower HRQoL, compared with controls (all *p* < 0.001). These differences remained statistically significant after adjustment for age, sex, and body mass index. Exploratory analyses revealed no statistically significant differences between diabetes subtypes, although subgroup comparisons were limited by sample size. Conclusions: Psychological distress is highly prevalent among Romanian adults with DM and is associated with poorer self-perceived health-related quality of life. The results support the relevance of incorporating systematic psychosocial assessment into routine diabetes care.

## 1. Introduction

Diabetes mellitus (DM) is a common chronic disease globally. It poses important public health challenges due to long-term complications, healthcare costs, and daily impacts on patients’ lives. DM also causes significant psychological discomfort. This discomfort can affect patients’ well-being, self-management, and perceived health. In diabetes research, psychological distress includes anxiety (worry or unease), depressive symptoms (ongoing sadness or loss of interest), and perceived stress (how stressful an individual finds life circumstances). Psychological distress is now seen as a key psychosocial aspect of diabetes care [1,2].

A large body of evidence indicates that adults with diabetes experience poorer health-related quality of life (HRQoL) than the general population. HRQoL is defined as the perceived physical, psychological, and social well-being associated with health. Anxiety and depression are the most common psychological comorbidities (co-occurring mental health conditions). Treatment demands, fear of complications, and lifestyle restrictions further lower the quality of life [2,3]. Early research shows that emotional suffering linked to diabetes management is central to the daily experience of diabetes [3].

To better describe this burden, the concept of diabetes-related psychological distress was introduced. This refers to emotional challenges directly tied to living with diabetes and helps distinguish these disease-specific responses from mood or anxiety disorders [4,5,6]. It reflects the cumulative emotional impact of ongoing self-care, worries about complications, and interactions with medical care systems. These often coexist with anxiety and depression and reduce patient-reported outcomes and HRQoL [6,7]. International recommendations now emphasize routine psychosocial assessment as part of comprehensive diabetes management [6].

Despite these advances, evidence remains uneven across regions. Data from Eastern European countries, including Romania, remains scarce. Differences in medical care delivery, access to mental health services, socioeconomic conditions, and cultural views of psychological symptoms may affect both the prevalence and expression of psychological distress in adults with diabetes. Findings from Western European or North American populations may not generalize directly to this region [8,9,10].

Both type 1 and type 2 diabetes lead to psychological challenges. However, comparative data between subtypes in adults is limited and inconsistent. An exploratory analysis of differences between subtypes may yield useful insights. These comparisons are limited by small sample sizes and clinical differences, so they require prudent evaluation [11,12].

Cross-sectional observational studies are appropriate for describing psychological distress and HRQoL at the population level. This is especially true when the main objective is to quantify group differences rather than infer causality. When combined with appropriate control groups and statistical adjustment for key confounders, this approach can generate clinically relevant data. Such data can aid future longitudinal research and the development of integrated care strategies.

### Study Rationale and Objectives

This study assessed anxiety, depressive symptoms, perceived stress, and health-related quality of life among Romanian adults with DM and healthy controls using validated tools. It also explored differences between diabetes subtypes. By providing region-specific data and focusing on effect sizes and adjusted comparisons, this study enhances understanding of the psychosocial burden of diabetes.

## 2. Materials and Methods

### 2.1. Study Design and Participants

This cross-sectional study compared psychological distress and health-related quality of life (HRQoL) in adults with DM and healthy controls. The study ran from August to October 2025 in Bucharest, Romania. Reporting followed the STROBE guidelines.

Participants were recruited using a consecutive convenience sampling approach from outpatient medical settings and community sources within the same geographical area. A total of 400 adults were enrolled, including 201 individuals with DM and 199 healthy controls.

Of those with diabetes, 38 participants (18.9%) had type 1 DM. The remaining 163 (81.1%) had type 2 or unspecified DM. The latter includes cases where the diabetes type of diabetes could not be determined from questionnaires or available medical documents.

#### Eligibility Criteria

Inclusion criteria for the diabetes group were:

- Age between 18 and 75 years;
- Confirmed diagnosis of DM for at least 12 months;
- Ability to complete self-administered questionnaires independently.

Exclusion criteria for all participants included:

- Current diagnosis of a major psychiatric disorder or neurological disease;
- Cognitive impairment interfering with questionnaire completion;
- Severe systemic illness or active malignancy;
- Refusal to participate.

Healthy controls came from the same area and met the same eligibility criteria. They also needed no self-reported history of DM, prediabetes, or other chronic metabolic disorders. Diabetes was excluded by taking a medical history.

### 2.2. Data Collection and Variables

Data came from standardized self-administered questionnaires and a structured clinical data sheet. A trained researcher supervised to ensure completeness and consistency.

#### 2.2.1. Demographic and Clinical Variables

Demographic variables included age, sex, educational level, marital status, and residence area (urban/rural).

For diabetes participants, clinical data included disease duration, body mass index (BMI; kg/m^2^), and treatment type. Treatment types were lifestyle measures, oral antidiabetic agents, insulin, or combined therapy.

For the control group, we checked if they had diabetes through a questionnaire and self-report; laboratory confirmation was not available.

#### 2.2.2. Psychological Assessment Instruments

All psychometric instruments used in the study have been previously validated and were administered in their Romanian-language versions.

1. State–Trait Anxiety Inventory (STAI-Y1 and STAI-Y2)
  Anxiety was measured using the State–Trait Anxiety Inventory, which has two parts: one for current feelings of anxiety (STAI-Y1) and one for general tendencies to feel anxious (STAI-Y2). Each part has 20 items. The scores were rescaled from 0 to 100 to facilitate comparisons across measures. This scaling does not affect how the results are ranked or their statistical interpretation. Higher scores mean greater anxiety. Both parts of the inventory were shown to be very reliable (Cronbach’s α > 0.90).
2. Beck Depression Inventory (BDI)
  Depressive symptoms were assessed using the 21-item BDI. Total scores range from 0 to 63, with higher scores reflecting greater depressive symptom severity.
3. Holmes–Rahe Stress Inventory (HR)
  We measured perceived stress with the Holmes–Rahe Stress Inventory. This tool adds up stressful life events. Scores above 300 indicate a high health risk related to stress.
4. EuroQol 5-Dimensions Visual Analogue Scale (EQ-5D VAS)
  Health-related quality of life was measured with the EQ-5D Visual Analogue Scale. Participants rated their health from 0 (worst imaginable) to 100 (best imaginable) [13]. Lower scores mean poorer perceived health.

### 2.3. Statistical Analysis

#### 2.3.1. Data Processing and Descriptive Statistics

Statistical analysis used IBM SPSS Statistics 26.0 (IBM Corp., Armonk, NY, USA). Results were cross-validated in Python 3.11 (SciPy 1.11). We report continuous variables as mean ± SD for normal data, or as median (interquartile range) for non-normal data. Categorical variables are given as frequencies and percentages.

#### 2.3.2. Normality Testing

Distribution normality was assessed using the Shapiro–Wilk test, and homogeneity of variances was evaluated with Levene’s test.

#### 2.3.3. Group Comparisons and Effect Sizes

To compare the diabetes and control groups, we used Welch’s *t*-test for normally distributed data. This test handles unequal group sizes. If needed, we used simpler, non-parametric tests.

For three-group comparisons (type 1 DM, type 2/unspecified DM, and controls), we used ANOVA with Tukey’s test for normal data. We used the Kruskal–Wallis test with Bonferroni-adjusted comparisons for non-normal data.

We calculated effect sizes with Hedges’ g. An effect size of 0.20 to 0.49 is small, 0.50 to 0.79 is medium, and 0.80 or above. There were group differences in age and BMI, so we used statistical methods to adjust. We used ANCOVA and multiple linear regression, controlling for age, sex, and BMI. Results with and without these adjustments are shown. Results with and without these adjustments are provided.

#### 2.3.4. Correlation Analyses

Associations between psychological measures and clinical variables (age, BMI, disease duration) were examined using Pearson’s or Spearman’s correlation coefficients, as appropriate.

All statistical tests were two-tailed, and statistical significance was set at *p* < 0.05. Ninety-five percent confidence intervals (95% CI) were calculated where applicable.

### 2.4. Data Quality Assurance

Data were checked for completeness and logical consistency through double-entry verification. Outliers exceeding ±3 standard deviations were reviewed individually and excluded only if deemed clinically implausible. Statistical outputs were independently verified across analytical platforms.

### 2.5. Ethical Considerations

The study protocol was approved by the Ethics Committee of the University of Medicine and Pharmacy “Carol Davila”, Bucharest, Romania (protocol code no. 20158/12.08.2025; approval date: 12 August 2025). The study was conducted in accordance with the principles of the Declaration of Helsinki (2013 revision). All participants provided written informed consent prior to participation, and data confidentiality was maintained throughout data collection, analysis, and publication.

## 3. Results

### 3.1. Demographic and Clinical Characteristics

A total of 400 participants were included in the analysis, comprising 201 adults with DM and 199 healthy controls (Table 1). Among participants with diabetes, 38 (18.9%) were classified as having type 1 DM, while 163 (81.1%) were classified as having type 2 or unspecified DM, reflecting cases in which diabetes subtype could not be unequivocally determined based on questionnaire data and available medical documentation.

Participants with diabetes were significantly older than healthy controls (54.2 ± 12.1 vs. 45.1 ± 13.8 years, *p* < 0.001) and had a significantly higher body mass index (29.4 ± 5.8 vs. 25.1 ± 4.7 kg/m^2^, *p* < 0.001). Sex distribution did not differ significantly between groups (*p* = 0.11). No statistically significant differences were observed with respect to educational level, marital status, or area of residence (all *p* > 0.05). The mean duration of diabetes among affected participants was 9.8 ± 7.2 years.

These differences underscore the necessity of adjusting subsequent analyses for age and BMI.

### 3.2. Psychological Status and Health-Related Quality of Life: Diabetes Versus Controls

Unadjusted comparisons demonstrated statistically significant differences between participants with diabetes and healthy controls across all psychological distress measures and HRQoL (Table 2). Scores for state anxiety (STAI-Y1), trait anxiety (STAI-Y2), depressive symptoms (BDI), and perceived stress (Holmes–Rahe) were significantly higher within participants with diabetes, while EQ-5D VAS scores were significantly lower (all *p* < 0.001).

Effect size analysis indicated very large differences for anxiety measures (STAI-Y1: Hedges’ g = 3.40; STAI-Y2: g = 3.16) and a large difference for perceived stress (g = 1.26). Depressive symptoms showed a moderate effect size (g = 0.58), while the difference in HRQoL demonstrated a small-to-moderate effect (g = −0.36).

To account for potential confounding, multivariable analyses adjusting for age, sex, and BMI were performed. After adjustment, differences between the diabetes and control groups remained statistically significant for all psychological distress measures and HRQoL (all adjusted *p* < 0.01), indicating that the observed associations were not entirely attributable to demographic or anthropometric differences.

### 3.3. Psychological Distress and HRQoL Across Diabetes Subtypes

Three-group comparisons, including participants with type 1 diabetes, type 2/unspecified diabetes, and healthy controls, revealed significant overall group differences for anxiety, depressive symptoms, perceived stress, and HRQoL (Table 3).

Post hoc analyses demonstrated that both diabetes subgroups exhibited significantly higher psychological distress and lower HRQoL compared with healthy controls. No statistically significant differences were observed between the type 1 and type 2/unspecified diabetes subgroups across psychological or HRQoL measures. Due to the relatively small proportion of participants with type 1 diabetes and the heterogeneity of the type 2/unspecified group, these subgroup comparisons should be interpreted as exploratory.

### 3.4. Associations Between Psychological Distress and HRQoL

Correlation analyses demonstrated significant inverse associations between HRQoL (EQ-5D VAS) and measures of psychological distress. Moderate negative correlations were observed between EQ-5D VAS scores and state anxiety (r ≈ −0.48, *p* < 0.001), trait anxiety (r ≈ −0.46, *p* < 0.001), and depressive symptoms (r ≈ −0.55, *p* < 0.001). Higher perceived stress was also associated with lower HRQoL (r ≈ −0.42, *p* < 0.001).

After adjustment for age and BMI, psychological variables remained the strongest correlates of HRQoL, whereas no additional demographic or clinical variables demonstrated independent associations.

### 3.5. Supplementary Figures

Graphical representations of psychological distress and HRQoL measures across study groups are provided in the Supplementary Materials (Figures S1–S5). These figures visually support the statistical findings and illustrate consistent patterns of higher psychological distress and lower HRQoL among participants with diabetes compared with healthy controls.

### 3.6. Summary of Key Findings

In summary, adults with diabetes mellitus exhibited significantly higher levels of psychological distress and lower self-perceived health-related quality of life compared with healthy controls. These differences persisted after adjustment for age, sex, and body mass index. Anxiety and perceived stress demonstrated particularly large between-group effects, while depressive symptoms and HRQoL showed moderate and small-to-moderate effects, respectively. Exploratory analyses did not identify meaningful differences between diabetes subtypes.

## 4. Discussion

This cross-sectional study investigated psychological distress and HRQoL among adults with DM. Results show that people with DM have higher levels of state and trait anxiety, depressive symptoms, and perceived stress compare to control. They also report lower self-perceived HRQoL than healthy controls. These data were similar across diabetes subtypes and remained significant after adjusting for age, sex, and BMI. This indicates that psychological distress is a strong and clinically relevant aspect of DM, beyond demographic differences.

### 4.1. Psychological Distress, Anxiety, and Depression in Diabetes

The very large effect sizes for anxiety and the large effect size for perceived stress indicate a substantial psychological burden among adults with DM. The extremely large effect sizes for STAI scores (Hedges’ g > 3) show a near-separation of score distributions between groups. Importantly, the linear 0–100 rescaling of STAI scores cannot inflate standardized effect sizes (e.g., Hedges’ g). Both the mean difference and the standard deviation are scaled proportionally. Nevertheless, ceiling effects cannot be excluded due to the high mean anxiety scores in the diabetes group. Future studies should examine score distributions and potential saturation using additional anxiety instruments and clinically defined thresholds. Notably, similar findings have been reported across diverse clinical and sociocultural contexts, including periods of heightened stress such as the COVID-19 pandemic. During these times, anxiety and stress prevalence among individuals with diabetes exceeded 70% [14,15,16,17,18]. Taken together, these data suggest that anxiety and stress are highly prevalent features of DM rather than transient reactions linked to specific events.

Several authors have described a two-way link between psychological distress and metabolic problems [19,20,21]. While these links make biological sense, our study cannot establish causality. Our results should be seen as evidence that these conditions co-occur, not as evidence that one causes the other. Other research shows that long-term stress can affect hormone and immune systems tied to insulin resistance and blood sugar swings [22,23]. New molecular studies, like those on stress-related long non-coding RNAs, indicate a shared biology between emotional distress and metabolic issues [24]. These data serve as background but do not directly explain our study’s results.

Depressive symptoms were also more pronounced in participants with DM, showing a moderate effect size. This finding is consistent with research showing that diabetes and depression often occur together [25,26,27]. Earlier studies found that depression in people with diabetes relates to worse blood sugar control, lower adherence to treatment, and higher cardiovascular risk [28,29,30]. While these outcomes were not examined here, our results support that depressive symptoms are part of the psychosocial burden of diabetes.

### 4.2. Health-Related Quality of Life and Psychological Distress

Participants with diabetes reported significantly lower HRQoL, as reflected by EQ-5D VAS scores. Although the effect size was smaller than for anxiety or stress, it remains clinically important and in line with previous systematic reviews identifying emotional distress, treatment burden, and limited social support as key determinants of reduced HRQoL in diabetes [27,31,32,33,34].

Building on these findings, previous studies show that diabetes-related distress more strongly influences HRQoL than metabolic indicators alone, especially in chronic disease [35,36,37,38,39,40]. In Romanian cohorts, socioeconomic factors, educational attainment, and depression are independent predictors of impaired HRQoL in people with type diabetes [41]. Similarly, researchers have found comparable patterns in other populations, confirming that HRQoL is shaped by psychological and social factors [42,43,44,45].

Extending these observations, and in accordance with previous findings, we observed moderate inverse correlations between HRQoL and anxiety, depression, and perceived stress, confirming that psychological distress is closely linked to subjective health perception. Meta-analytic data show that improvements in emotional well-being are associated with better self-management and enhanced quality of life in diabetes [46]. However, these results do not prove that psychological interventions alone improve HRQoL, as we did not evaluate interventional effects.

### 4.3. Biological and Oxidative Stress Pathways: Contextual Evidence

Many studies suggest that oxidative stress and neuroendocrine dysregulation may link psychological distress and metabolic disease [47,48,49]. Excessive reactive oxygen species and stress-induced hyperglycemia are associated with adverse cardiovascular and metabolic outcomes [48,49]. These findings give valuable biological context. However, they are outside the scope of the current study and should be seen as background, not direct confirmation. Future work should combine psychological assessments with molecular and metabolic biomarkers to further explore these pathways.

### 4.4. Psychological Distress Across Diabetes Subtypes

We did not find significant differences in anxiety, depression, stress, or HRQoL between type 1 and type 2/unspecified diabetes. This suggests that psychological distress may be a common issue for all people with diabetes, not just those with a specific type. Earlier studies also report high stress and emotional burden in both groups, though the causes of distress may vary [50,51]. Our subgroup findings are exploratory due to the small number of participants with type 1 diabetes and the diversity within the type 2/unspecified group.

### 4.5. Clinical and Public Health Implications

The findings emphasize the need to integrate psychosocial assessment into routine DM care. Many people with diabetes experience undetected anxiety or depression [52]. Validated tools, such as the STAI, BDI, and EQ-5D, help identify psychological distress early.

Integrated, multidisciplinary care combining medical and psychological support improves patient outcomes [28,43]. This study does not assess intervention effectiveness, so it supports comprehensive care models rather than specific therapies. Address socioeconomic disparities and care access, as health inequalities impact psychological and metabolic outcomes [41,42].

### 4.6. Regional Relevance and Future Directions

In Romania and Eastern Europe, diabetes rates rise, but mental health services remain limited [53,54]. The end of COVID-19 in mid-2023 presents a chance to strengthen integrated care. Add psychosocial screening to diabetes management to improve patient outcomes [55,56]. Future research should integrate clinical, psychological, and biological markers to clarify links between emotional distress and diabetes outcomes.

## 5. Strengths and Limitations

This study’s relatively large sample (*n* = 400) provided adequate statistical power for group comparisons. The study thoroughly assessed psychological distress and health-related quality of life (HRQoL). We measured state and trait anxiety, depressive symptoms, perceived stress, and HRQoL with well-validated psychometric instruments (STAI-Y1/Y2, BDI, Holmes–Rahe Stress Inventory, and EQ-5D VAS), enhancing reliability and comparability. Hedges’ g effect sizes clarified the magnitude of group differences with statistical significance.

Several limitations should also be acknowledged. First, the cross-sectional design precludes causal inference regarding the temporal relationships between psychological distress, HRQoL, and DM. Second, although participants were categorized by diabetes subtype, the type 2/unspecified diabetes group included cases without complete phenotypic characterization, potentially limiting the precision of subtype-specific comparisons. Third, the absence of diabetes in the control group was based on structured medical history and self-report rather than laboratory confirmation, which may have led to minimal misclassification. Fourth, HRQoL was assessed using the EQ-5D VAS, a global measure that does not capture disease-specific quality-of-life domains; the inclusion of diabetes-specific instruments such as the Diabetes-Specific Quality of Life (DSQOL) or the Audit of Diabetes-Dependent Quality of Life (ADDQoL) could have provided more nuances.

Reliance on self-reported questionnaires may lead to recall bias (where participants inaccurately remember information) and mood-dependent responses (answers influenced by a participant’s current emotional state). Clinical indicators such as glycemic control (measured by blood hemoglobin A1c, or HbA1c), diabetes-related complications (medical problems directly linked to diabetes), and hypoglycemia frequency (how often low blood sugar episodes occur) were not included in the statistical models. Without these biomedical parameters, we cannot explore how metabolic status interacts with psychological distress.

## 6. Future Research

Future studies should use prospective studies to determine the timing and mechanisms by which psychological distress, health-related quality of life, and diabetes outcomes interact. Researchers are recommended to integrate psychological assessments with biomedical markers, such as glycemic control, complication burden, and treatment types, to more clearly define the relationship between emotional stress and metabolic regulation.

Further research needs to use both diabetes-specific and general quality-of-life tools. Larger, well-defined samples by diabetes subtype will allow comprehensive subgroup analyses. Region-specific studies of integrated psychosocial care models can help find ways to reduce the psychological burden of diabetes in Romania and Eastern Europe.

## 7. Conclusions

In conclusion, this cross-sectional study shows that adults with DM have much higher levels of psychological distress, including anxiety, depressive symptoms, and perceived stress, as well as lower self-perceived health-related quality of life compared to healthy controls. Since the study is cross-sectional, these links do not prove causality. Both types of diabetes were included, which suggests that psychosocial burden is common in DM as a chronic condition, though differences between subtypes should be viewed with caution.

The results show a strong connection between emotional distress and health in adults with diabetes. Because this is a cross-sectional study, we cannot say one causes the other. The findings highlight that psychological factors play an important role in the burden of diabetes. Regularly checking psychological well-being can help better understand patient-reported outcomes in this group.

More long-term and intervention studies are needed to better understand how these factors are related over time and to see if integrated approaches that address both psychological and clinical aspects of DM management are helpful, especially in areas with limited mental health services.

These findings should be interpreted in light of the observational study design and in the lack of objective metabolic markers.

## Figures and Tables

**Table 1:** Demographic and clinical characteristics of the study participants.

Variable	Diabetes (*n* = 201)	Healthy Controls (*n* = 199)	*p*-Value
Age (years), Mean ± SD	54.2 ± 12.1	45.1 ± 13.8	<0.001
Sex (female), *n* (%)	111 (55.2%)	97 (48.7%)	0.11
BMI (kg/m^2^), Mean ± SD	29.4 ± 5.8	25.1 ± 4.7	<0.001
Education (secondary +), *n* (%)	176 (87.6%)	175 (88.0%)	0.89
Marital status (married), *n* (%)	135 (67.2%)	127 (63.8%)	0.53
Urban residence, *n* (%)	145 (72.1%)	155 (77.9%)	0.18
Duration of diabetes (years), Mean ± SD	9.8 ± 7.2	—	—

Legend: Values are presented as mean ± standard deviation (SD) for continuous variables and as number (percentage) for categorical variables. *p*-values refer to comparisons between participants with diabetes mellitus and healthy controls and were calculated using Welch’s independent-samples *t*-test for continuous variables and the chi-square (χ^2^) test for categorical variables, as appropriate. BMI, body mass index.

**Table 2:** Psychological distress and health-related quality of life in participants with DM and healthy controls.

Variable	Diabetes (Mean ± SD)	Controls (Mean ± SD)	*t* (df)	*p*-Value	Hedges’ *g*
State anxiety (STAI-Y1)	84.9 ± 12.9	41.9 ± 12.4	48.18 (797.4)	<0.001	+3.40
Trait anxiety (STAI-Y2)	83.8 ± 13.0	44.0 ± 12.2	44.69 (796.1)	<0.001	+3.16
Depression (BDI)	14.7 ± 11.4	9.0 ± 8.0	8.19 (719.0)	<0.001	+0.58
Perceived stress (Holmes–Rahe)	445.7 ± 192.8	230.8 ± 143.5	17.89 (740.9)	<0.001	+1.26
Quality of life (EQ-5D VAS)	66.4 ± 33.8	76.4 ± 19.6	−3.62 (321.7)	0.0003	−0.36

Legend: Values are presented as mean ± standard deviation (SD). STAI-Y1 and STAI-Y2 scores are reported on the 0–100 transformed scale, as described in the Methods section. Group comparisons were performed using two-tailed Welch’s independent-samples *t*-tests. Effect sizes are reported as Hedges’ *g*, corrected for unequal sample sizes. Positive values of *g* indicate higher scores in the diabetes group, whereas negative values indicate lower scores compared with healthy controls. HRQoL, health-related quality of life; EQ-5D VAS, EuroQol 5-Dimensions Visual Analogue Scale.

**Table 3:** Psychological distress and health-related quality-of-life across diabetes subtypes and healthy controls.

Variable	Test	F/H	*p*-Value	Significant Post Hoc Pairs (*p* < 0.05)
State anxiety (STAI-Y1)	ANOVA	25.6	<0.001	Controls < Type 1 DM; Controls < Type 2/unspecified DM
Trait anxiety (STAI-Y2)	ANOVA	31.2	<0.001	Controls < Type 1 DM; Controls < Type 2/unspecified DM
Depression (BDI)	Kruskal–Wallis	45.9	<0.001	Controls < Type 1 DM; Controls < Type 2/unspecified DM
Perceived stress (HR)	Kruskal–Wallis	18.4	0.002	Controls < Type 1 DM; Controls < Type 2/unspecified DM
Quality of life (EQ-5D VAS)	ANOVA	7.32	0.008	Controls > Type 1 DM; Controls > Type 2/unspecified DM

Legend: Overall group differences were assessed using one-way analysis of variance (ANOVA) for normally distributed variables and the Kruskal–Wallis test for non-normally distributed variables. *F* values correspond to ANOVA results, and *H* values correspond to Kruskal–Wallis tests. Post hoc pairwise comparisons were performed using Tukey’s honestly significant difference test or Bonferroni-adjusted Mann–Whitney U tests, as appropriate. Analyses comparing diabetes subtypes were exploratory. DM, diabetes mellitus; HRQoL, health-related quality of life; EQ-5D VAS, EuroQol 5-Dimensions Visual Analogue Scale.

## Data Availability

The original contributions presented in this study are included in the article/Supplementary Material. Further inquiries can be directed to the corresponding authors.

## Supplementary Materials

The following supporting information can be downloaded at: https://www.mdpi.com/article/10.3390/healthcare14020158/s1, Figure S1: State anxiety (STAI-Y1) scores across study groups; Figure S2: Trait anxiety (STAI-Y2) scores across study groups; Figure S3: Depressive symptoms (BDI) across study groups; Figure S4: Perceived stress (Holmes–Rahe) scores across study groups; Figure S5: Health-related quality of life (EQ-5D VAS) across study groups.

## Abbreviations

The following abbreviations are used in this manuscript:

ADDQoL	Audit of Diabetes-Dependent Quality of Life
BDI	Beck Depression Inventory
BMI	body mass index
DD	diabetes-related distress
DM	diabetes mellitus
DSQOL	diabetes-specific quality of life
EQ-5D VAS	EuroQol 5-Dimensions Visual Analogue Scale
GDM	gestational diabetes mellitus
HR	Holmes–Rahe Stress Inventory
HRQoL	health-related quality of life
ROS	reactive oxygen species
QoL	quality of life
T1D	type 1 diabetes
SD	standard deviation
STAI	State–Trait Anxiety Inventory
VAS	visual analogue scale
