# Supplementary material for: Psychological Distress and Health-Related Quality of Life in Romanian Adults with Diabetes Mellitus: A Cross-Sectional Study
Source: Healthcare (Basel). 2026 Jan 8;14(2):158. doi: 10.3390/healthcare14020158 (PMC12841257; doi:10.3390/healthcare14020158)
Supplement: Supplementary file 1 [file healthcare-14-00158-s001.zip › healthcare-4028651-supplementary.pdf]

Supplementary Materials

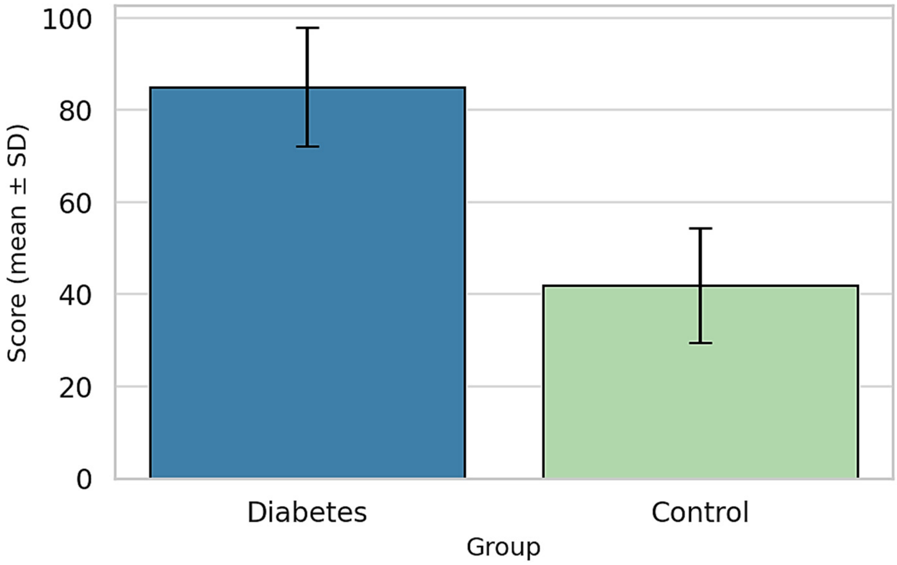

Figure S1. State anxiety (STAI-Y1) scores across study groups.

Legend: Bar plot illustrating mean State–Trait Anxiety Inventory state anxiety (STAI-Y1) scores with standard deviations for participants with type 1 diabetes mellitus, type 2/unspecified diabetes mellitus, and healthy controls. STAI-Y1 scores are presented on the 0–100 transformed scale.

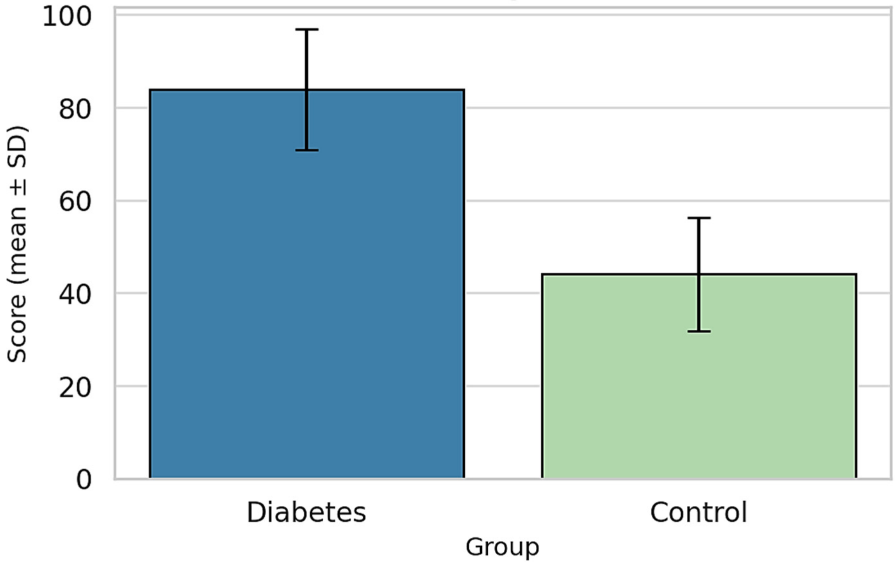

Figure S2. Trait anxiety (STAI-Y2) scores across study groups.

Legend: Bar plot illustrating mean State–Trait Anxiety Inventory trait anxiety (STAI-Y2) scores with standard deviations for participants with type 1 diabetes mellitus, type 2/unspecified diabetes mellitus, and healthy controls. STAI-Y2 scores are presented on the 0–100 transformed scale.

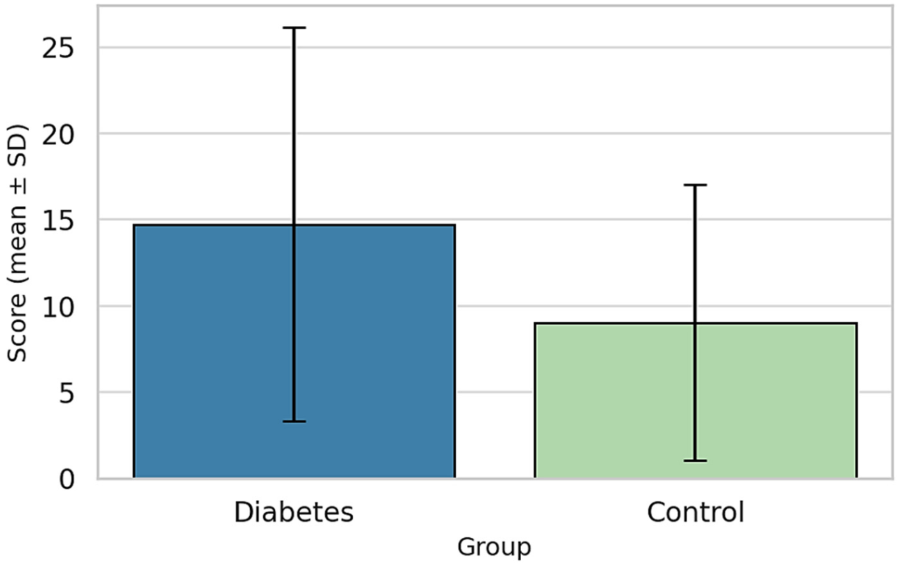

Figure S3. Depressive symptoms (BDI) across study groups.

Legend: Bar plot illustrating mean Beck Depression Inventory (BDI) scores with standard deviations for participants with type 1 diabetes mellitus, type 2/unspecified diabetes mellitus, and healthy controls.

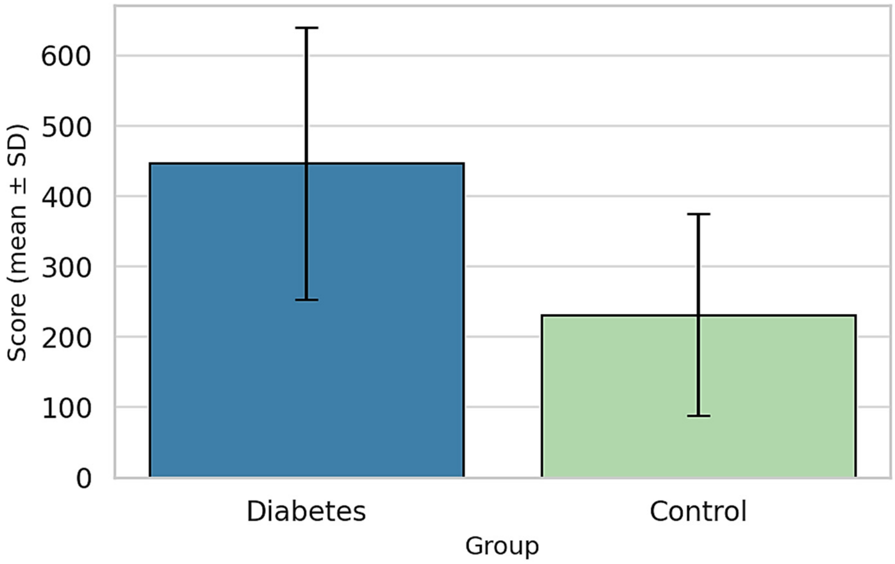

Figure S4. Perceived stress (Holmes–Rahe) scores across study groups.

Legend: Bar plot illustrating mean Holmes–Rahe stress scores with standard deviations for participants with type 1 diabetes mellitus, type 2/unspecified diabetes mellitus, and healthy controls.

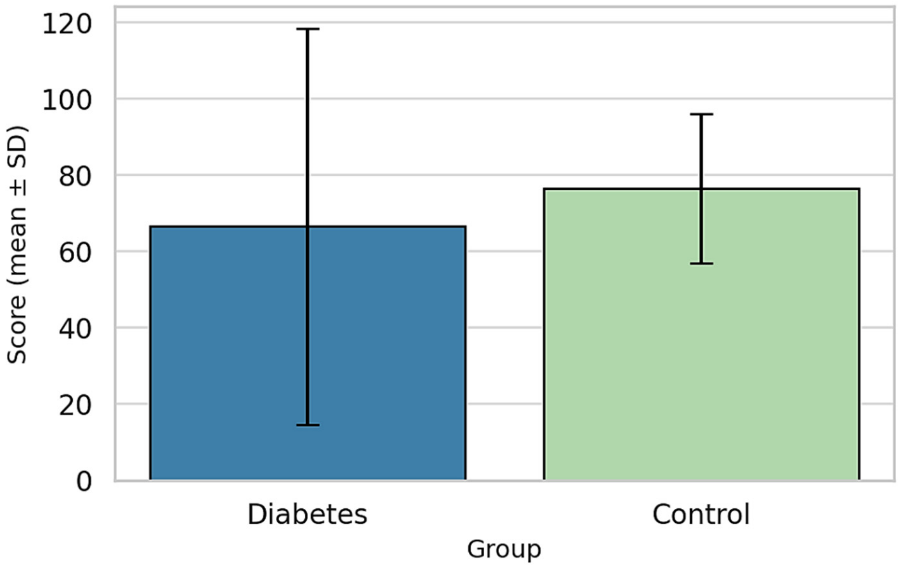

Figure S5. Health-related quality of life (EQ-5D VAS) across study groups.

Legend: Bar plot illustrating mean EuroQol 5-Dimensions Visual Analogue Scale (EQ-5D VAS) scores with standard deviations for participants with type 1 diabetes mellitus, type 2/unspecified diabetes mellitus, and healthy controls.
